# Antigen-presenting genes and genomic copy number variations in the Tasmanian devil MHC

**DOI:** 10.1186/1471-2164-13-87

**Published:** 2012-03-12

**Authors:** Yuanyuan Cheng, Andrew Stuart, Katrina Morris, Robyn Taylor, Hannah Siddle, Janine Deakin, Menna Jones, Chris T Amemiya, Katherine Belov

**Affiliations:** 1 Faculty of Veterinary Science, University of Sydney, Sydney, NSW, Australia; 2 Genome Resource Center, Benaroya Research Institute at Virginia Mason, Seattle, WA, USA; 3 Department of Primary Industries, Parks, Water and Environment, Prospect, TAS, Australia; 4 Department of Pathology, University of Cambridge, Cambridge, UK; 5 Research School of Biology, The Australian National University, Canberra, ACT, Australia; 6 School of Zoology, University of Tasmania, Hobart, TAS, Australia; 7 Faculty of Veterinary Science, University of Sydney, RMC Gunn B19, Sydney, NSW 2006, Australia

**Keywords:** MHC, Tasmanian devil, Copy number variation, Devil facial tumour disease

## Abstract

**Background:**

The Tasmanian devil (*Sarcophilus harrisii*) is currently under threat of extinction due to an unusual fatal contagious cancer called Devil Facial Tumour Disease (DFTD). DFTD is caused by a clonal tumour cell line that is transmitted between unrelated individuals as an allograft without triggering immune rejection due to low levels of Major Histocompatibility Complex (MHC) diversity in Tasmanian devils.

**Results:**

Here we report the characterization of the genomic regions encompassing MHC Class I and Class II genes in the Tasmanian devil. Four genomic regions approximately 960 kb in length were assembled and annotated using BAC contigs and physically mapped to devil Chromosome 4q. 34 genes and pseudogenes were identified, including five Class I and four Class II loci. Interestingly, when two haplotypes from two individuals were compared, three genomic copy number variants with sizes ranging from 1.6 to 17 kb were observed within the classical Class I gene region. One deletion is particularly important as it turns a Class Ia gene into a pseudogene in one of the haplotypes. This deletion explains the previously observed variation in the Class I allelic number between individuals. The frequency of this deletion is highest in the northwestern devil population and lowest in southeastern areas.

**Conclusions:**

The third sequenced marsupial MHC provides insights into the evolution of this dynamic genomic region among the diverse marsupial species. The two sequenced devil MHC haplotypes revealed three copy number variations that are likely to significantly affect immune response and suggest that future work should focus on the role of copy number variations in disease susceptibility in this species.

## Background

**Figure 1:**
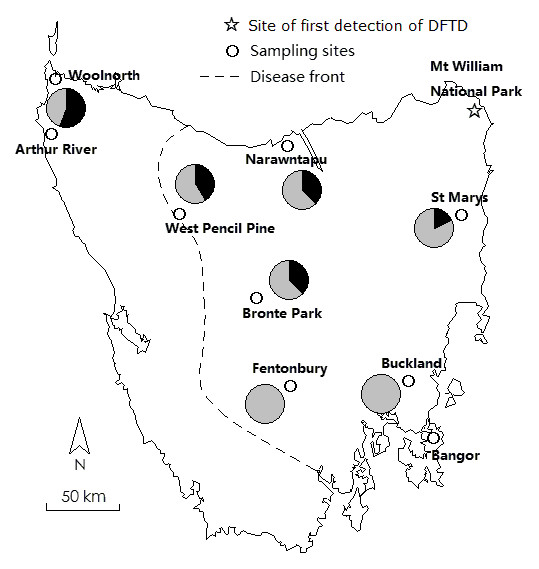
**Map of Tasmania showing the sampling sites, location of the first citing of DFTD in 1996 (Mt William), and the present location of the disease front**. The black segment in the pie charts shows the proportion of individuals with a deletion at *Saha-UA*, and the grey segment shows the proportion with intact *Saha-UA *gene.

The Tasmanian devil (*Sarcophilus harrisii*), an endemic species on the island state of Tasmania, Australia, is the largest remaining carnivorous marsupial in the world [1]. Tasmanian devils ("devils" for short) were found on mainland Australia up to 3,000 to 4,000 years ago [2]. The Tasmanian population has been isolated for over 12,000 years and has undergone two population crashes, due to the current disease epidemic and in around 1900 [3]. As a result, the devil population has an overall low level of genetic diversity [4]. Currently, the devil faces extinction due to the emergence of a fatal contagious cancer - Devil Facial Tumour Disease (DFTD). DFTD was first detected in 1996 at Mt William National Park in the northeast of Tasmania (Figure 1) [3]. Since then, it has rapidly spread south and westwards to over 85% of the original devil distributional range and caused severe population declines [5]. Research has revealed that DFTD is a clonal cell line, likely of Schwann cell origin, which is transmitted between individuals as an allograft by cellular inoculation (e.g. biting) [6,7]. The "grafted" tumour cells can overcome immunological barriers of the host and adapt in unrelated individuals without inducing immune rejection [8]. The low level of genetic diversity at the Major Histocompatibility Complex (MHC) is believed to have contributed to this process [9].

The MHC is one of the most studied gene regions in vertebrates due to its critical roles in disease resistance and transplantation success. MHC genes have been cloned and characterized from representatives of all vertebrate classes except agnathan fish [10]. Based on the structure and function of their encoded proteins, MHC genes are grouped into three classes (Class I, II and III) [11]. Class I and II genes are further classified by function as classical and nonclassical, with classical Class I and II genes encoding cell surface molecules that present antigens to T lymphocytes [11]. The MHC Class I genes are encoded by an α chain, which associates with a β_2_-microglobulin chain to become a functional Class I molecule [10]. Classical Class I (Class Ia) molecules are ubiquitously expressed in all tissue types and function in the recognition and destruction of foreign, virus-infected or malignant cells by cytotoxic T cells [12]. Nonclassical Class I (Class Ib) genes encode Class I-like molecules with varied functions, and generally exhibit lower expression levels, tissue specific expression and/or lower levels of polymorphism [13]. MHC Class II molecules are heterodimers of an α chain and a β chain, both of which are encoded in the MHC. They are expressed in cells participating in immune responses such as B lymphocytes, dendritic cells and macrophages. These molecules present antigens derived from intravesicular and extracellular pathogens to CD4 helper T cells, which release signals to trigger antibody production and inflammatory responses that kill the pathogens [14]. In most species, there are multiple closely related MHC gene paralogues, which result from gene duplication events during the evolution of MHC [15,16]. Due to selective pressures from ever-changing pathogens in the environment, these antigen-presenting MHC genes evolve rapidly and are usually highly polymorphic in their peptide-binding regions (exon 2 and 3 of Class I genes; exon 2 of Class II genes), enabling the immune system to recognize an extensive range of pathogenic antigens [17].

Previously, we isolated and characterized devil MHC Class I and Class II β chain transcripts [18] and demonstrated low levels of genetic variation in these sequences, especially in eastern Tasmania [19]. In the northwest, a slightly higher level of diversity was observed using single-strand conformation polymorphism (SSCP) typing, while sequencing revealed variation in Class I allelic numbers in different individuals [19], but due to the inherent difficulties in studying the MHC in non-model species, we were not able to rule out null alleles or experimental artefacts. Here we describe the MHC Class I and Class II regions of the Tasmanian devil based on the construction of BAC contigs. We confirm that variation in the number of Class I genes occurs due to a deletion within a Class Ia locus.

## Results

### Characterization of MHC regions

The MHC regions of two individuals were characterized. Cedric, whose parents came from DFTD-free northwestern Tasmania (father from Arthur River and mother from Woolnorth; Figure 1), produced an antibody response when injected with irradiated DFTD cells (Alex Kreiss, University of Tasmania, personal communication) and at the beginning of the project was thought to be a "resistant" animal because he did not develop DFTD following a disease challenge. Since then Cedric has succumbed to DFTD, but remains one of very few animals to be able to mount an antibody response to DFTD. The other individual, named Spirit, was originally from Bangor (Figure 1) and was euthanized in 2008 due to multiple DFTD lesions and metastases to the lungs.

**Figure 2:**
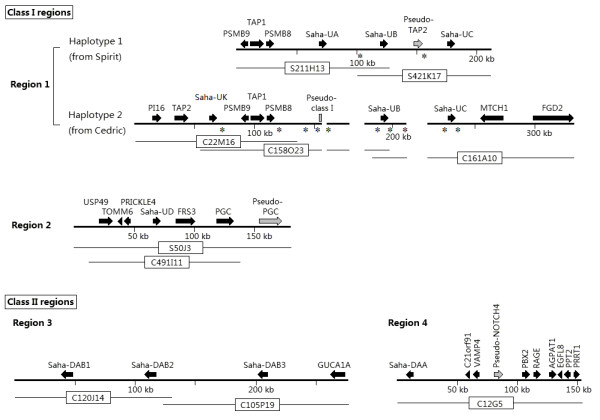
**Schematic diagram of Tasmanian devil genomic regions containing MHC genes**. Arrows represent annotated genes. BAC clones used for sequence assembly are indicated by lines below the annotation. For the Class I Region 1, alignment of two haplotypes is shown, as significant differences are found between them. The three gaps within haplotype 2 (and BAC C158O23 and C161A10) represent three segments that are not present in this haplotype as compared to haplotype 1. Asterisks indicate positions of 12 putative LINE fragments.

Four genomic regions of approximately 960 kb in length were assembled and annotated based on ten fully sequenced BAC clones (seven from Cedric and three from Spirit) (Figure 2). 34 genes and pseudogenes were predicted, comprising five Class I genes, four Class II genes, four antigen-processing genes, eight Class III genes and thirteen other genes and pseudogenes. GenBank accession numbers of the BAC clones and coordinates of the predicted genes are shown in Table 1. The annotated genes were mostly named after their orthologues in the human with the exceptions of MHC Class I and II genes, which were given species-specific names based on the nomenclature proposed by Klein and colleagues [20] and their evolutionary relationships with previously characterized marsupial genes [21,22].

**Table 1:** List of annotated BAC clones.

BAC	Accession	Start	End	Strand	Description
C22M16	FQ482140	24985	22341	-	Proteasome subunit, beta type, 8
		
		42720	30519	-	Transporter 1, ATP-binding cassette, sub-family B
		
		43296	46824	+	Proteasome subunit, beta type, 9
		
		67202	64208	-	MHC Class I antigen Saha-UK
		
		98510	88331	-	Transporter 2, ATP-binding cassette, sub-family B
		
		125729	116120	-	Peptidase inhibitor 16-like

C158O23	FQ482137	98067	99377	+	MHC Class I pseudogene

C161A10	FQ482138	97718	77164	-	Mitochondrial carrier homolog 1
		
		122296	155379	+	FYVE, RhoGEF and PH domain containing 2

S211H13	FQ482144	67289	70233	+	MHC Class I antigen Saha-UA
		
		118698	121642	+	MHC Class I antigen Saha-UB

S421K17	FQ482146	67403	64222	-	Transporter 2, ATP-binding cassette, sub-family B pseudogene
		
		36094	33148	-	MHC Class I antigen Saha-UC

C491I11	FQ482142	8066	18154	+	Ubiquitin specific peptidase 49
		
		26396	24778	-	Translocase of outer mitochondrial membrane 6
		
		32637	30374	-	Prickle homolog 4 (Drosophila)
		
		53046	56295	+	MHC Class I antigen Saha-UD
		
		70180	88114	+	Fibroblast growth factor receptor substrate 3
		
		105924	114685	+	Progastricsin (pepsinogen C)

S50J3	FQ482147	154766	172999	+	Progastricsin (pepsinogen C) pseudogene

C120J14	FQ790236	47684	38237	-	MHC Class II DA β chain 1
		
		119765	110505	-	MHC Class II DA β chain 2

C105P19	FQ790235	83441	74884	-	MHC Class II DA β chain 3
		
		148648	135902	-	Guanylate cyclase activator 1A

C12G5	FQ790241	13651	9537	-	MHC Class II DA α chain
		
		60582	59696	-	Protein EURL homolog
		
		73613	71308	-	Vesicle-associated membrane protein 4-like
		
		84027	87750	+	NOTCH4 pseudogene
		
		108416	112090	+	Pre-B-cell leukemia homeobox 2
		
		114221	117186	+	Advanced glycosylation end product-specific receptor
		
		127655	131100	+	1-acylglycerol-3-phosphate O-acyltransferase 1
		
		135753	133198	-	Epidermal growth factor-like protein 8
		
		141212	138220	-	Palmitoyl-protein thioesterase 2
		
		148877	150447	+	Proline-rich transmembrane protein 1

Genes in the BAC overlapping regions are only shown once. Letters C and S in BAC clone names indicate Cedric's and Spirit's library, respectively

**Figure 3:**
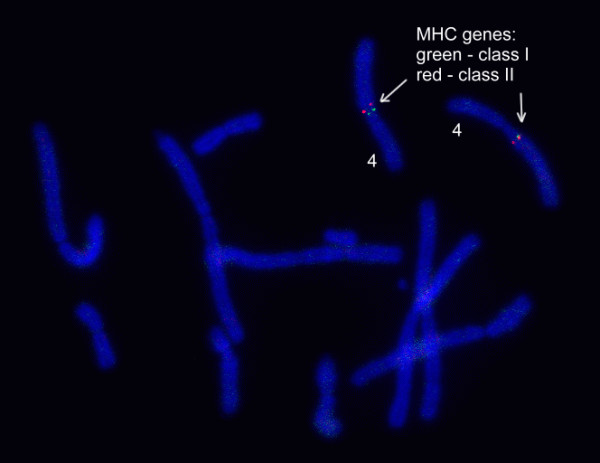
**FISH image showing genomic locations of Tasmanian devil MHC Class I and II genes**.

**Figure 4:**
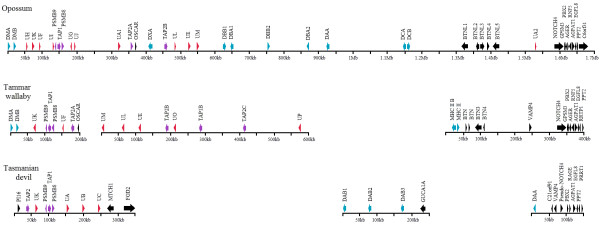
**Comparison of MHC regions containing Class I or II genes between Tasmanian devil, grey short-tailed opossum **[21]**and tammar wallaby **[35]. Class I, II and antigen-processing genes are represented with red, blue and purple arrows, respectively. Class II regions of tammar wallaby are not shown due to high complexity.

Two of the analysed regions contain Class I genes (Region 1 and 2) and the other two contain Class II (Region 3 and 4) (Figure 2). All four regions were physically mapped to the same area on devil Chromosome 4q, indicating the genomic location of the devil MHC (see Figure 3 for Region 1 and 3, and Additional file 1: Figure S1 for Region 2 and 4). Based on comparisons to the MHC of the grey short-tailed opossum (*Monodelphis domestica*; herein referred to as "opossum") and the tammar wallaby (*Macropus eugenii*), Region 3 is likely located between Region 1 and Region 4, which encompasses a suite of well-conserved Class III genes (Figure 4). In Region 2, Class I *Saha-UD *is flanked by five non-MHC genes *USP49, TOMM6, PRICKLE4, FRS3 *and *PGC*, which are found ~12 MB proximal from the MHC in human and ~16 Mb distal from the MHC in opossum; however, according to FISH mapping result, these genes are closer to the MHC in the devil than in opossum (Additional file 1: Figure S1).

### Class I genes

**Figure 5:**
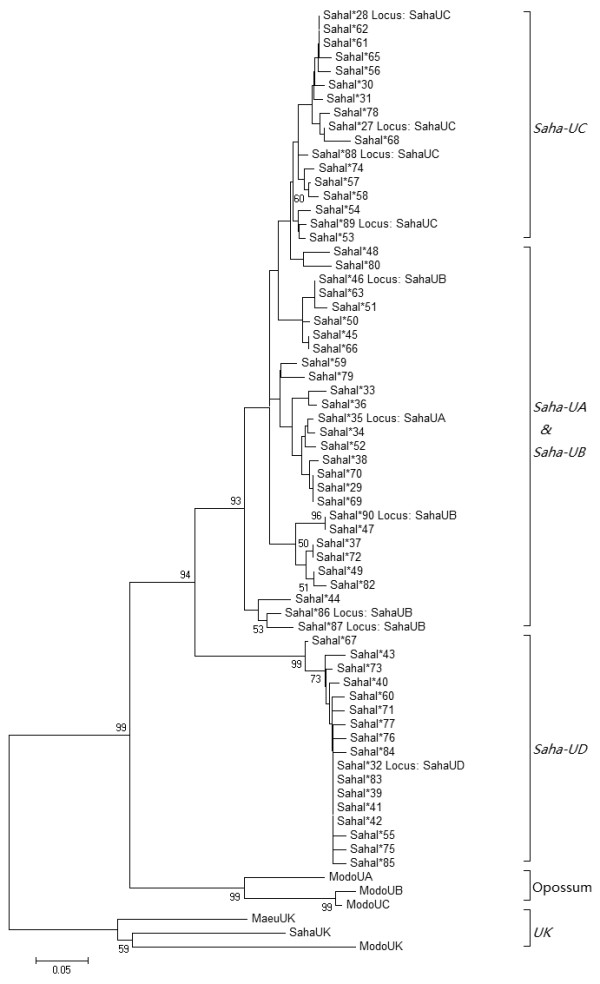
**Phylogenetic analysis of Tasmanian devil MHC Class I sequences variants**. The phylogenetic relationship was inferred using the Neighbour-Joining method [46]. The percentage of replicate trees in which the associated sequences clustered together in the bootstrap test (1,000 replicates) are displayed next to the branches, indicating the level of reliability of the phylogeny [47]. Bootstrap frequencies lower than 50% are not shown. Phylogenetic analysis was conducted in MEGA5 [45]. *Modo-UK *[GenBank:EU886686] and *Maeu-UK *[GenBank:CU463018] are orthologous *UK *genes in the grey short-tailed opossum and tammar wallaby. *Modo-UA *[GenBank:DQ067089], *UB *[GenBank:NM_001079820] and *UC *[GenBank:NM_001079819] are Class I genes in the opossum.

A total of five Class I genes were identified and named *Saha-UA*, *UB*, *UC*, *UD *and *UK*. Except *Saha-UD*, they are all closely linked (Region 1 in Figure 2) and are localised with *PSMB8*, *PSMB9*, *TAP1 *and *TAP2 *genes, which are involved in antigen processing and transportation. *Saha-UA*, *UB*, *UC *and *UD *have evolved from recent gene duplications in the devil lineage (shown in Figure 5) and are not orthologous to Class I genes in the opossum or the tammar wallaby. *Saha-UK *is orthologous to *Modo-UK *in the opossum and *Maeu-UK *in the tammar wallaby (Figure 5), both of which have been suggested to be nonclassical genes [22,23].

The genomic data allowed us to assign MHC alleles to loci using phylogenetics (Figure 5, Additional file 2: Figure S2). Table 2 describes the alleles sequenced on each of the two haplotypes, and by deduction the alleles found on the un-sequenced haplotypes of Cedric and Spirit. We were able to resolve that previously isolated devil Class I sequence variants SahaI*27 and *28*, *35*, *46*, and *32 *belong to locus *Saha-UC*, *UA*, *UB *and *UD*, respectively. Meanwhile, five novel alleles, named SahaI*86 - 90, were found and assigned to genes. Alleles from *Saha-UD*, which were previously called group 2 alleles [19], are divergent from other genes and form a discrete phylogenetic clade with 94% bootstrap support. *Saha-UA*, *UB *and *UC *are closely related, with *UA *and *UB *alleles interspersed within the same clade. At nucleotide level, the sequence identity between these three genes is extremely high (> 97.7%), with *Saha-UA *and *UB *sharing up to 99% nucleotide identity in their introns (Table 3).

**Table 2:** MHC Class I haplotypes of two model Tasmanian devils - Cedric and Spirit

Class Ia loci	Cedric's haplotypes		Spirit's haplotypes	
	a (BAC sequenced)	b	a (BAC sequenced)	b
*Saha-UA*	-	-	SahaI*35	SahaI*35
*Saha-UB*	SahaI*86	SahaI*87	SahaI*46	SahaI*90
*Saha-UC*	SahaI*88	SahaI*89	SahaI*28	SahaI*27

**Table 3:** Comparison of Tasmanian devil MHC Class Ia genes.

		Sequence identity		
		
Gene	Length (bp)	*Saha-UA*	*Saha-UB*	*Saha-UC*
*Saha-UA*	2921	-	99.0%	97.8%
*Saha-UB*	2921	98.9%	-	97.7%
*Saha-UC*	2923	98.3%	98.3%	-

Sequence identity in exons (shown below the diagonal) and introns (shown above the diagonal) are calculated separately

**Table 4:** Comparison of Tasmanian devil MHC DAB genes.

			Sequence identity	
		
Gene	Length (bp)	*Saha-DAB1*	*Saha-DAB2*	*Saha-DAB3*
*Saha-DAB1*	9448	-	95.7%	61.9%
*Saha-DAB2*	9261	96.9%	-	62.8%
*Saha-DAB3*	8558	95.3%	96.1%	-

Sequence identity in exons (shown below the diagonal) and introns (shown above the diagonal) are calculated separately Sequence identity

**Figure 6:**
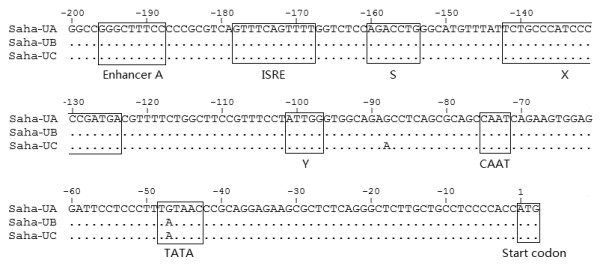
**Putative promoter elements of Tasmanian devil MHC Class Ia genes**. The boxed sequences indicate putative sites of an enhancer A, an interferon stimulated response element (ISRE), the S-X-Y motifs and the CAAT and TATA boxes.

The sequence 200 bp upstream of the translation start sites was searched for gene regulatory elements, as promoters of MHC Class I genes in mammals are mostly contained within this range [24]. In *Saha-UA*, *UB *and *UC*, this region is highly similar, with only one single nucleotide variation in the TATA sites. Putative sites of an enhancer A, an interferon stimulated response element (ISRE), the S-X-Y motifs, CAAT and TATA boxes were identified (Figure 6). *Saha-UD *was not included in the alignment in Figure 6 due to low sequence identity. Compared to *Saha-UA*, *UB *and *UC*, regulatory elements including enhancers, ISRE, S and X motifs are not conserved at this locus, which may indicate different expression or function of this gene.

### Comparison of two MHC class I haplotypes

The MHC region containing the Class Ia genes in Cedric and Spirit (Region 1 in Figure 2) contains three indels. Spirit's haplotype has an intact *Saha-UA *gene, whereas Cedric's has a deletion (~1646 bp in size) that results in the loss of a large portion (from exon 2 to intron 5) of *Saha-UA *and renders it a pseudogene. Furthermore, Cedric's haplotype also lacks two other long segments that are present in Spirit's, one (~12230 bp) lying between the pseudogene and *Saha-UB*, and the other (~16970 bp) between *Saha-UB *and *Saha-UC*. Further investigation on this genomic region revealed 12 putative Long INterspersed Element (LINE) segments interspersing the MHC genes (Figure 2), nine of which closely flank (within 2 kb) the indels and *Saha-UA*, *UB *and *UC*. Two of the putative LINEs are located inside the indels and only found in Spirit's haplotype.

**Figure 7:**
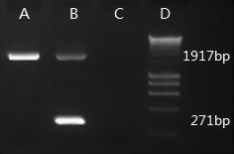
**Gel image showing two types of Tasmanian devil MHC Class I haplotypes**. Gel lanes: A - Spirit's type with intact Class I gene *Saha-UA*; B - Cedric's type with a deletion in *Saha-UA*; C - negative control; D - HyperLadder I (Bioline).

A PCR test was developed to detect the presence or absence of the deletion in *Saha*-*UA *in devils. Haplotypes without the deletion generate a single band of 1917 bp in size, which contains amplicons from *Saha-UA*, *UB *and *UC *genes. A second band (271 bp) is generated when a haplotype contains the deletion in *Saha-UA*, representing an amplicon from only the pseudogene, which has arisen due to the deletion (Figure 7).

### Population study

The frequency of the *Saha-UA *deletion was assessed in samples from seven geographic areas across Tasmania (see pie charts in Figure 1). 72 individuals were investigated: 25 from DFTD-affected areas in eastern Tasmania (five from each of St Marys, Narawntapu, Bronte Park, Buckland and Fentonbury); 12 from West Pencil Pine, an area on the disease front; and 35 from the DFTD-free northwestern coast region. The deletion is most prevalent in the northwest (found in 54.8% of tested individuals), followed by West Pencil Pine (41.7%), Bronte Park (40%), Narawntapu (40%) and St Marys (20%). None of individuals from Fentonbury and Buckland contained the deletion.

### Class II genes

**Figure 8:**
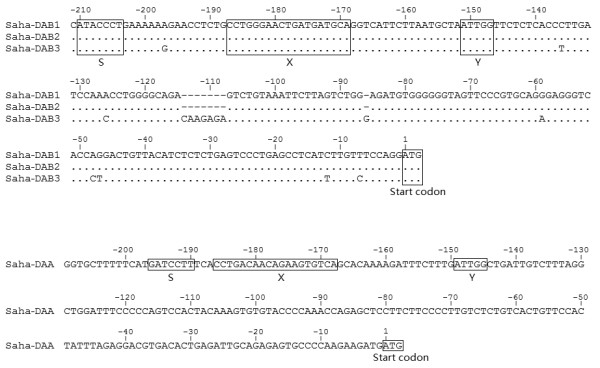
**Upstream sequences of Tasmanian devil MHC Class II β chain and α chain genes**. The boxed sequences indicate putative sites of S-X-Y motifs.

Four Class II genes were identified, all belonging to the marsupial Class II *DA *gene family. Previously isolated DAB transcripts were aligned against the three β chain paralogues and 100% match was found between transcripts SahaDAB*01 [GenBank:EF591102], SahaDAB*03 [GenBank:EF591104] and SahaDAB*05 [GenBank:EF591105] and genes *Saha-DAB1*, *DAB2 *and *DAB3*, respectively. Sequence comparison of these three genes is shown in Table 4. *Saha-DAB1 *and *DAB2 *share high nucleotide identity in both exons (96.9%) and introns (95.7%). They are both similar to *Saha-DAB3 *in exon sequences, but differ from it significantly in the introns due to multiple deletions/insertions of nucleotide fragments. In the 5' untranslated region, putative sites of S-X-Y motifs are identified in both α and β chain genes, though TATA and CAAT elements are not found in these genes (Figure 8).

## Discussion

### Function of MHC genes

Based on the genomic characterization of the MHC genes, as well as expression and diversity studies, we are now in the position to assign previously identified MHC alleles to loci and make informed deductions about the likely role of these genes in immune response.

Four Class II loci are described in this study, *Saha-DAA*, *DAB1*, *DAB2 *and *DAB3*. Transcripts from the three β chain genes have previously been described [18]. These loci likely encode functional antigen-presenting molecules based on homology to other members of the *DA *gene family, which have been studied in a variety of marsupial species [reviewed in [25]]. No other Class II gene families have been identified in the devil, indicating that either the devil only has one functional Class II gene family or the other genes are too divergent from known marsupial Class II genes to be detected by the probes.

**Figure 9:**
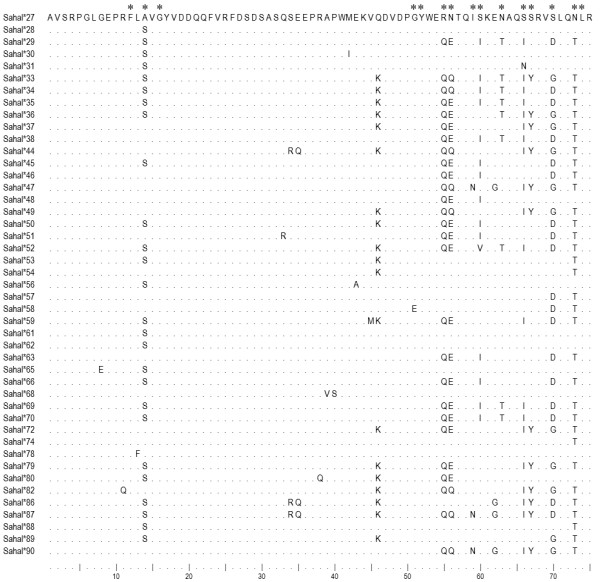
**Amino acid alignment of partial α1 domain of *Saha-UA, UB *and *UC *alleles**. Asterisks indicate putative peptide-binding sites in a Class I molecule [48].

Amongst the five characterized Class I loci, we propose that *Saha-UA*, *UB *and *UC *are functional Class Ia genes on the basis of three facts. First, their transcripts have been amplified from all tissue types examined so far, including blood, spleen, skin, liver, kidney and DFTD cells [9,18]. Second, genetic variation, though the level is not high, is present at these loci, particularly in the putative peptide-binding regions (Figure 9). Third, promoter elements in the 5' untranslated region are well conserved between these genes and the Class Ia genes in the opossum and the tammar wallaby [22], sharing 91.8% and 83.6% nucleotide identity respectively. Whether the single nucleotide variation in TATA box affects the expression level of *Saha-UA *remains uncertain. The number of Class Ia loci in the devil is comparable to those in the opossum (one) and the tammar wallaby (three).

*Saha-UK *has been found transcribed in the blood and spleen and is likely a Class Ib gene based on orthology with the Class Ib *Modo-UK *in the opossum and *Maeu-UK *in the tammar wallaby. It has been suggested that the high conservation of this gene over extended evolutionary periods indicates that it may serve a critical, marsupial-specific function [22].

**Figure 10:**
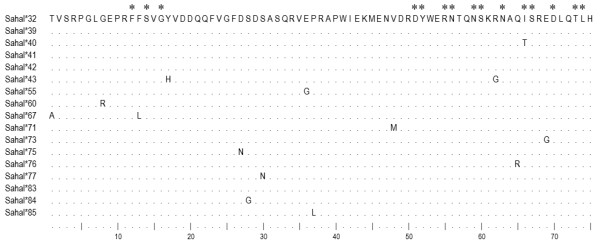
**Amino acid alignment of partial α1 domain of *Saha-UD *alleles**. Asterisks indicate putative peptide-binding sites in a Class I molecule [48].

*Saha-UD*, which has been found transcribed in the blood, spleen and DFTD cells, shows features of a Class Ib gene. This locus has significantly lower levels of polymorphism than *Saha-UA*, *UB *and *UC*, and its 5' regulatory elements are divergent. Within the α1 domain, *Saha-UD *alleles share extremely high sequence identity (> 97.7%) and only one of the 15 putative peptide-binding amino acid residues is polymorphic (Figure 10). Elucidation of the functional role of *Saha-UD *in the devil remains to be determined.

Genomic characterization allowed us to assign previously characterized "group 1" alleles to Class Ia loci *Saha-UA*, *UB *and *UC*, and "group 2" alleles to *Saha-UD *([19], Figure 5). The previous description of individuals containing only "group 1" or "group 2" alleles [19] was likely due to experimental artefact caused by nucleotide mismatches (two in *Saha-UA*, *UB *and *UC*; four in *Saha-UD*) within the PCR primer hybridisation sites. Here, we propose that Class Ia loci *Saha-UB *and *UC *are likely present in all devils, whereas *Saha-UA *is lost in certain haplotypes. We suggest that in future studies new primers should be designed for *Saha-UD *and the three Class Ia genes separately to ensure high primer efficiency.

### Copy number variations (CNVs) in the MHC

A genomic CNV is a duplication or deletion of a genomic segment larger than 1 kb in size [26]. In addition to single nucleotide polymorphisms, CNVs represent a major class of genetic variation and are widespread in the human genome [27,28]. Large-scale genome-wide disease association studies have revealed a number of CNVs in the HLA that are associated with Crohn's disease, rheumatoid arthritis and type 1 diabetes [26,29].

When we first proposed the hypothesis of "Class I gene copy number variation" in the devil, we were referring to the concept of CNV in a narrow sense, namely variation in the number of Class I loci [19]. With the new findings from this study, it is necessary to expand the concept to take into account other CNVs of non-coding sequences lying within the devil MHC.

Three genomic copy number variants that intersperse with the MHC Class Ia genes were identified between Cedric's and Spirit's haplotypes. The one within *Saha-UA *is convincingly a deletion, while the other two are difficult to classify as either duplications or deletions. The loss of functional Class I genes due to CNVs is not specific to the devil. A case has been reported for a human haplotype where a 4 kb deletion turns *HLA-A *into a pseudogene [30]. The mechanisms underlying the generation of these CNVs in the devil MHC are not clear, though a possible explanation may be implied from the presence of the putative LINE fragments in the affected regions encompassing the three CNVs and *Saha-UA*, *UB *and *UC *genes (Figure 2). These retrotransposons may play a role in causing the CNVs as evidence has been found in primate genomes that LINEs are centrally involved in the generation of CNVs and can mediate deletions up to 18 kb in size [31,32].

The higher frequency of the *Saha-UA *deletion in the northwest may indicate that the eastern and western populations have been exposed to different selective pressures. It is tempting to continue to speculate that the deletion of *Saha-UA*, which was found in Cedric and occurs in high frequency in the northwest of Tasmania, may provide animals with some level of resilience to DFTD. This is consistent with the observation that the spread of DFTD is slowing down as the disease front reaches genetically disparate populations in the northwest and that the incidence of disease in these populations is much lower than in similar populations in the east [33]. This gene deletion may be advantageous for the following reasons. First, the loss of *Saha-UA *may increase the MHC antigenic dissimilarity between host and tumour, as a *Saha-UA *allele, SahaI*35, has been found to be transcribed in the tumour [19]. Second, the CNVs may alter the expression level of the adjacent MHC genes by affecting regulatory elements such as promoters and enhancers as well as inhibitory elements. In fact, it has been found that CNVs can even influence the expression of genes that are up to 1 Mb away [34]. Here we have only looked at two devil MHC haplotypes and have only scratched the surface of the genetic variation that could be present amongst devil genomes. The role of this variation in the varied susceptibility/resistance of devils to DFTD needs to be further investigated.

### Comparison with the opossum and tammar wallaby MHC

Previous comparative studies revealed that the MHC of the opossum and the tammar wallaby exhibit distinct features in gene content and organisation [21,35]. Sequencing of Tasmanian devil MHC regions has provided us a better understanding of the marsupial MHC.

In the tammar wallaby, all Class Ia genes (*UA, UB *and *UC*) are un-linked to the MHC region [22], whereas in the opossum the only confirmed Class Ia gene (*UA1*) is located within the MHC and close to antigen-processing genes ([21], Figure 4). The organisation of Class Ia genes in the devil is similar to that of the opossum, with *UA, UB *and *UC *adjacent to *TAP1, TAP2, PSMB9 *and *PSMB8*. This indicates that the dispersal of Class I genes across the genome in the tammar wallaby is not a common characteristic of the Australasian marsupial and has occurred after the divergence of devil and tammar wallaby's common ancestor at ~66 million years before present [36].

Four Class II gene families have been characterized in the opossum - *DA, DB, DC *and *DM *[21]. *DA*, *DB *and *DM *have also been identified in the tammar wallaby. The Class II genes have undergone large-scale expansion in the tammar wallaby, resulting in up to 10 *DAB *loci [35]. However, such an expansion of Class II genes is not seen in the devil, with devils having a single *DAA *and three *DAB *genes. On the contrary, the devil genome may have undergone gene deletions that have lead to loss of the *DB *and *DC *gene families.

In this study, we have focused on characterizing MHC Class Ia and II genes in the devil. Future work will involve characterization of the functional roles of the putative Class Ib genes, as well as genes involved in the antigen processing machinery.

## Conclusions

Four Tasmanian devil genomic regions containing five MHC Class I genes and four MHC Class II genes were characterized by BAC based sequencing, which allowed us to assign previously sequenced MHC alleles to loci. We propose that *Saha-UA*, *UB *and *UC *are Class Ia genes, *Saha-UK *is a transcribed Class Ib gene and the role of *Saha-UD *remains to be determined. The expression of *Saha-UA*, *UB *and *UC *may be influenced by three genomic CNVs that are found within or adjacent to these loci. Future studies should focus on the role of CNVs in the MHC in susceptibility/resistance of devils to DFTD.

## Methods

### Construction of bacterial artificial chromosome (BAC) libraries

Two BAC libraries were produced. The first one, designated VMRC-49, was constructed from whole blood genomic DNA of Cedric (three-year-old male). The second library, designated VMRC-50, was constructed from genomic DNA extracted from the liver of Spirit (two-year-old male). The genomes of both animals were recently sequenced [37]. The genome assembly was not able to provide an accurate picture of the MHC, which contains multiple closely related genes that have arisen from recent gene duplications. Therefore, a BAC clone sequencing project was required.

The BAC libraries were produced in the Genome Resource Center at the Benaroya Research Institute at Virginia Mason, Seattle, USA. Detailed procedures of library construction have been described previously [38,39]. The quality of the DNA was checked by running a pulsed field gel electrophoresis (PFGE) on a CHEF-DR^® ^III system (BioRad). The DNA was partially digested in an *EcoRI/EcoRI-methylase *competition reaction and size fractionated by analytical PFGE on a BioRad CHEF Mapper^® ^XA system. DNA fragments from the appropriate size fraction were ligated into the CopyControl™ pCC1BAC™ vector from Epicentre Technologies and transformed into Invitrogen ElectroMAX™ DH10B™ T1 Phage-Resistant *E. coli *cells. Transformants were arrayed into 384-well LB/chloramphenicol/glycerin microtiter plates (Genetix) using colony-picking robots (Norgren Systems) and subsequently gridded onto 22 × 22 cm high-density nylon filters with a Total Array System (BioRobotics Ltd.). The average insert size was estimated to be ~140 kb. A total of 672 microtiter plates, which contain 258,048 BAC clones, and 14 high-density filters were produced for library VMRC-49. Assuming the size of the devil genome is similar to that of the opossum genome, which is around 3.6 Gb, this library will represent 10x coverage of the devil genome. For library VMRC-50, 432 plates containing 165,888 BAC clones and nine high-density filters were generated, estimated to represent 6.5x whole genome coverage.

### Characterization of MHC-positive BAC clones

#### MHC probes

MHC Class I and Class II β chain probes for library screening were designed based on devil cDNA sequences [18]. Two Class I probes were used to screen both libraries. The first one was a 274 bp fragment from Class I gene exon 2, which was amplified using PCR primers and conditions described previously [9]. The second Class I probe was a 191 bp fragment from exon 4, amplified using forward primer 5' -CAGTGCCGGGCCCAGGACTTTTA -3' and reverse primer 5' -CCCTCGTGCTGAACTCGGCAGGT -3'. PCR was carried out on devil genomic DNA in a total volume of 25 μl, which contains 1x High Fidelity Buffer (Invitrogen) consisting of 60 mM Tris-HCl (pH 8.9) and 18 mM (NH_4_)_2_SO_4_, 2.5 mM MgSO_4_, 0.2 mM each dNTP, 0.8 μM each primer, and 1.5 U of Platinum *Taq *DNA Polymerase High Fidelity (Invitrogen). PCR amplifications were performed on a BioRad MJ Mini Personal Thermal Cycler at the following conditions: 100°C hot lid; 94°C initial denaturation for 3 min; 32 cycles of 94°C denaturation for 30 sec, 60°C annealing for 30 sec, 72°C extension for 30 sec; and 72°C final extension for 10 min. Library VMRC-49 was also screened with two Class II probes for β chain and α chain genes. The β chain probe was a 237 bp fragment from devil *DAB *gene exon 3, amplified with forward primer 5' - AGCCCGAGGTGACTGTGTATC -3' and reverse primer 5'- CGTGGCAGGTGTAGACATCTC -3'. PCR conditions were same as above. The α chain probe was designed from the exon 2 of a tammar wallaby *DAA *gene ([GenBank:CU464025], position 141572-141757) and amplified from tammar wallaby genomic DNA using the same PCR reagents and conditions as described above. All PCR amplicons were isolated by running a 1.8% agarose gel using HyperLadder IV (Bioline) as size marker, and purified from the gel using MoBio UltraClean 15 DNA Purification Kit.

#### Library screening

Radioactively labelled probes were synthesized from approximately 50 ng of PCR amplified MHC gene fragments with either [α-^32^P]dCTP or [α-^32^P]dATP (PerkinElmer) using Random Primed DNA Labeling Kit from Roche Applied Science. Unincorporated dNTPs were removed from the probe with Illustra ProbeQuant G-50 Micro Columns from GE Healthcare. Hybridisation of BAC filters with MHC probes was carried out overnight at 60°C in Amersham Rapid-hyb™ Buffer (GE Healthcare). Excess background and non-specific binding was minimized by washing the filters at 60°C with a series of buffers (2x SSC, 2x SSC with 0.1% SDS, 1x SSC with 0.1% SDS, and 0.5x SSC with 0.1% SDS). Amersham Hyperfilm MP (GE Healthcare) was used to visualize positive clones after one to three days of exposure to the filters.

#### BAC clone sequencing

BAC DNA of the positive clones was purified from 100 ml LB/chloramphenicol bacterial culture using QIAGEN Large-Construct Kit. BACs containing unique MHC Class I or II genes were confirmed by direct end sequencing of BAC DNA with MHC primers, using standard sequencing service at the Australian Genome Research Facility Ltd. Fingerprinting analysis and complete sequencing of MHC-positive BAC clones was conducted at the Wellcome Trust Sanger Institute, Cambridge, UK. Traditional Sanger sequencing method was employed to ensure high assembly accuracy of paralogous MHC genes.

#### BAC sequence annotation

BAC sequences were aligned with human genomic and transcript sequences, non-human reference RNA sequences, and known or predicted opossum and tammar wallaby transcripts using web-based BLAST programs [40]. Genes were annotated manually based on the best BLAST hits and in accordance with the recommendations of the Human and vertebrate analysis and annotation guidelines [41]. Overlapping BAC sequences were identified and aligned using BLASTN and ClustalW programs [42]. A wallaby LINE-1 segment [GenBank:DQ275763] was used to search for putative LINE segments.

### Fluorescent *in situ *hybridisation (FISH)

BAC clones containing MHC Class I or II genes were physically mapped to a male devil karyotype following the protocol described previously by Alsop and colleagues [43]. Approximately 1 mg of BAC DNA was used to produce probes that were labelled by nick translation with either SpectrumOrange dUTP or SpectrumGreen dUTP (Abbott Molecular Inc.). Labelled probes were hybridised overnight to devil chromosomes, which were denatured for 1 min 40 sec. Slides were washed once at 60°C in 0.4x SSC with 0.3% Tween20 for 2 min and then once in 2x SSC with 0.1% Tween20 for 30 sec at room temperature. Chromosomes were counterstained in DAPI and mounted in VECTASHIELD^® ^Mounting Medium from Vector Laboratories Inc. A Zeiss Axioplan2 epifluorescence microscope was used to visualize the fluorescent signals. Images of DAPI stained metaphase chromosomes and fluorescent signals were captured using a SPOT RT Monochrome CCD charge-coupled device camera (Diagnostic Instruments Inc.) and merged using IP Lab imaging software (Scanalytics Inc.).

### Sequencing and analysis of MHC class I alleles

To test the Class I gene number variation hypothesis, it was necessary to ensure that all Class I loci in both Cedric and Spirit were characterized. To do this, we isolated and sequenced all Class I alleles from both individuals and assigned them to genes. We amplified exon 2 of the Class I genes from genomic DNA using protocols developed by Siddle and colleagues [9] with modifications in the reverse primer sequence (5'- CTCGCTCTGGTTGTAGTAGCC 3'). Two independent PCRs were performed for each individual. The PCR amplicons were gel-purified and cloned in a pGEM-T Easy Vector (Promega)/JM109 High Efficiency Competent Cells (Promega) cloning system. 32 positive clones were picked for each individual and plasmids were extracted using QIAGEN DirectPrep 96 MiniPrep Kit on a QIAvac Multiwell vacuum manifold (QIAGEN). Plasmids were sequenced with T7 primer at the Australian Genome Research Facility, Sydney, Australia. Sequences were quality-checked using Sequencher 4.1.4 (Gene Codes) and aligned with previous identified devil Class I alleles [19] in BioEdit 7.0.9 [44]. To minimize errors yielded during PCR, cloning and sequencing, new sequence variants were determined to be real alleles only if they were found in more than one PCR amplification. Alleles were assigned to genomic loci based on nucleotide sequence similarity. Evolutionary relationships of devil Class I sequences were analysed by constructing a Neighbour-Joining phylogenetic tree with MEGA5 [45].

### PCR primer design

Two pairs of new PCR primers were designed using program Oligo 6.7 (Molecular Biology Insights). One pair (forward 5'- TTTGCAAGCTTCCATGTCTCT -3', reverse 5'-CACTTGTGCTTGGAGTTCAGA -3') amplified partial exon 2 to exon 3 of the Class I gene *Saha-UK *from devil cDNA. The other pair was designed to detect a deletion in Class I gene *Saha-UA*, with the forward primer 5'- TGTCCCCCCCTCCGTCTCAG -3'binding to the end of intron 1 and the reverse 5'- CAGGAGAGGAGACCACACTAAGAT -3'binding to the end of intron 5. In about 5% of samples, this set of primers was found to work less efficiently, which could be due to nucleotide variations in primer binding sites. PCR conditions for both primer sets were the same as the one provided above. PCR products were sequenced to confirm correct amplification sites.

## Authors' contributions

YC, AS, KM, RT constructed the BAC libraries under the supervision of CTA. YC carried out all laboratory experiments, analysed the data and wrote the first draft of the manuscript. HS provided supervision in the lab. JD supervised FISH experiments. MJ provided devil samples. KB conceived and designed the project and revised the manuscript together with YC. All authors read drafts of the manuscript.

## Supplementary Material

Additional file 1 **Figure S1 FISH image showing genomic locations of BAC clone C12G5 and C491I11**. The individual used in this slide has a balanced translocation between Chr.1 and Chr.3. Click here for file

Additional file 2 **Figure S2 Alignment of sequences used to generate Figure **5**, including all Tasmanian devil Class I alleles identified so far**. Click here for file
