# Supplementary figures and images for: Antigen-presenting genes and genomic copy number variations in the Tasmanian devil MHC
Source: BMC Genomics. 2012 Mar 12;13:87. doi: 10.1186/1471-2164-13-87 (PMC3414760; doi:10.1186/1471-2164-13-87)

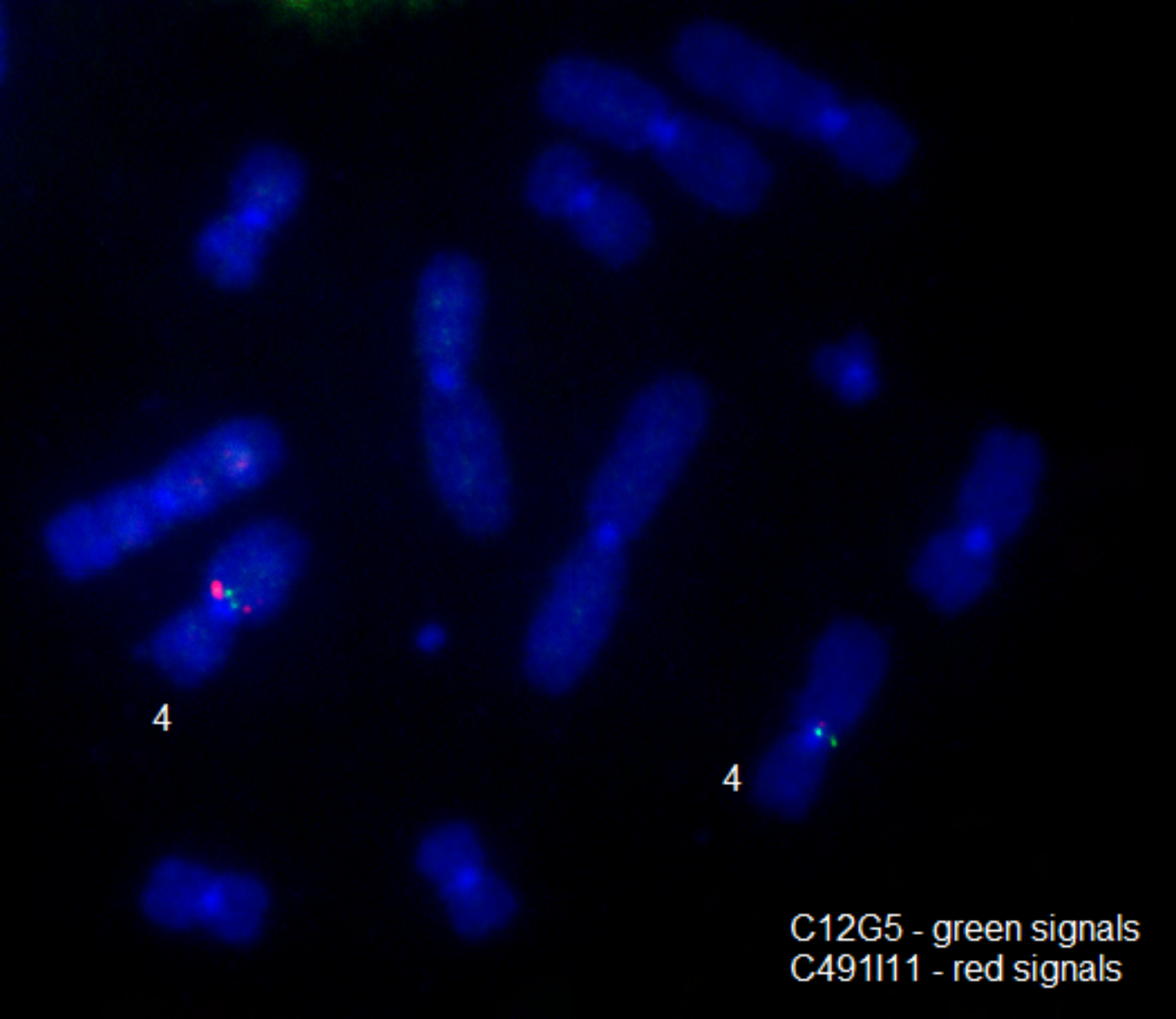

4

4

C12G5 - green signals  
C49111 - red signals

Supplement: Additional file 1 — Figure S1 FISH image showing genomic locations of BAC clone C12G5 and C491I11. The individual used in this slide has a balanced translocation between Chr.1 and Chr.3. [file 1471-2164-13-87-S1.PDF]
