# Supplementary material for: Antigen-presenting genes and genomic copy number variations in the Tasmanian devil MHC
Source: BMC Genomics. 2012 Mar 12;13:87. doi: 10.1186/1471-2164-13-87 (PMC3414760; doi:10.1186/1471-2164-13-87)
Supplement: Additional file 2 — Figure S2 Alignment of sequences used to generate Figure 5, including all Tasmanian devil Class I alleles identified so far. [file 1471-2164-13-87-S2.PDF]

>SahaI*86 Locus: SahaUB
GCCGTGTCCCGGCCCGGGCTCGGGGAGCCGCGGTTCCTCTCCGTGGGCTACGTGGACGAT
CAGCAGTTCGTGCGCTTCGACAGCGACAGCGCGAGTCAGAGGCAGGAGCCGCGGGCGCCG
TGGATGGAGAAGGTGAAGGACGTGGACCCGGGATACTGGGAGCGGAACACACAGATCAGT
AAGGGGAATGCACAGATTTACCGAGTGGACCTGCAGACCCTGCGC
>SahaI*87 Locus: SahaUB
GCCGTGTCCCGGCCCGGGCTCGGGGAGCCGCGGTTCCTCTCCGTGGGCTACGTGGACGAT
CAGCAGTTCGTGCGCTTCGACAGCGACAGCGCGAGTCAGAGGCAGGAGCCGCGGGCGCCG
TGGATGGAGAAGGTGAAGGACGTGGACCCGGGATACTGGGAGCAGCAGACACAGAACAGT
AAGGGGAATGCACAGATTTACCGAGTGGACCTGCAGACCCTGCGC
>SahaI*88 Locus: SahaUC
GCCGTGTCCCGGCCCGGGCTCGGGGAGCCGCGGTTCCTCTCCGTGGGCTACGTGGACGAT
CAGCAGTTCGTGCGCTTCGACAGCGACAGCGCGAGTCAGAGTGAGGAGCCGCGGGCGCCG
TGGATGGAGAAGGTGCAGGACGTGGACCCGGGATACTGGGAGCGGAACACACAGATCAGT
AAGGAGAACGCACAGAGTTCCCGAGTGAGCCTGCAGACCCTGCGC
>SahaI*89 Locus: SahaUC
GCCGTGTCCCGGCCCGGGCTCGGGGAGCCGCGGTTCCTCTCCGTGGGCTACGTGGACGAT
CAGCAGTTCGTGCGCTTCGACAGCGACAGCGCGAGTCAGAGTGAGGAGCCGCGGGCGCCG
TGGATGGAGAAGGTGAAGGACGTGGACCCGGGATACTGGGAGCGGAACACACAGATCAGT
AAGGAGAACGCACAGAGTTCCCGAGTGGGCCTGCAGACCCTGCGC
>SahaI*32 Locus: SahaUD
ACCGTGTCCCGGCCCGGACTCGGGGAGCCGCGATTCTTCTCCGTGGGCTACGTGGACGAT
CAGCAGTTCGTGGGCTTCGACAGCGACAGTGCGAGTCAGAGGGTGGAGCCGCGGGCACCA
TGGATAGAGAAGATGGAGAATGTGGACCGGGACTACTGGGAGCGGAACACGCAGAACAGT
AAGAGGAATGCACAAATTTCCCGAGAGGACCTGCAGACCCTACAC
>SahaI*35 Locus: SahaUA
GCCGTGTCCCGGCCCGGGCTCGGGGAGCCGCGGTTCCTCTCCGTGGGCTACGTGGACGAT
CAGCAGTTCGTGCGCTTCGACAGCGACAGCGCGAGTCAGAGTGAGGAGCCGCGGGCGCCG
TGGATGGAGAAGGTGAAGGACGTGGACCCGGGATACTGGGAGCAGGAGACACAGATCATT
AAGGAGACTGCACAGATTTCCCGAGTGGACCTGCAGACCCTGCGC
>SahaI*90 Locus: SahaUB
GCCGTGTCCCGGCCCGGGCTCGGGGAGCCGCGGTTCCTCGCCGTGGGCTACGTGGACGAT
CAGCAGTTCGTGCGCTTCGACAGCGACAGCGCGAGTCAGAGTGAGGAGCCGCGGGCGCCG
TGGATGGAGAAGGTGCAGGACGTGGACCCGGGATACTGGGAGCAGCAGACACAGAACAGT
AAGGGGAATGCACAGATTTACCGAGTGGGCCTGCAGACCCTGCGC
>SahaI*46 Locus: SahaUB
GCCGTGTCCCGGCCCGGGCTCGGGGAGCCGCGGTTCCTCGCCGTGGGCTACGTGGACGAT
CAGCAGTTCGTGCGCTTCGACAGCGACAGCGCGAGTCAGAGTGAGGAGCCGCGGGCGCCG
TGGATGGAGAAGGTGCAGGACGTGGACCCGGGATACTGGGAGCAGGAGACACAGATCATT
AAGGAGAACGCACAGAGTTCCCGAGTGGACCTGCAGACCCTGCGC
>SahaI*27 Locus: SahaUC
GCCGTGTCCCGGCCCGGGCTCGGGGAGCCGCGGTTCCTCGCCGTGGGCTACGTGGACGAT
CAGCAGTTCGTGCGCTTCGACAGCGACAGCGCGAGTCAGAGTGAGGAGCCGCGGGCGCCG
TGGATGGAGAAGGTGCAGGACGTGGACCCGGGATACTGGGAGCGGAACACACAGATCAGT
AAGGAGAACGCACAGAGTTCCCGAGTGAGCCTGCAGAACCTGCGC
>SahaI*28 Locus: SahaUC
GCCGTGTCCCGGCCCGGGCTCGGGGAGCCGCGGTTCCTCTCCGTGGGCTACGTGGACGAT
CAGCAGTTCGTGCGCTTCGACAGCGACAGCGCGAGTCAGAGTGAGGAGCCGCGGGCGCCG
TGGATGGAGAAGGTGCAGGACGTGGACCCGGGATACTGGGAGCGGAACACACAGATCAGT
AAGGAGAACGCACAGAGTTCCCGAGTGAGCCTGCAGAACCTGCGC
>SahaI*29
GCCGTGTCCCGGCCCGGGCTCGGGGAGCCGCGGTTCCTCTCCGTGGGCTACGTGGACGAT
CAGCAGTTCGTGCGCTTCGACAGCGACAGCGCGAGTCAGAGTGAGGAGCCGCGGGCGCCG
TGGATGGAGAAGGTGCAGGACGTGGACCCGGGATACTGGGAGCAGGAGACACAGATCATT
AAGGAGACTGCACAGATTTCCCGAGTGGACCTGCAGACCCTGCGC
>SahaI*30
GCCGTGTCCCGGCCCGGGCTCGGGGAGCCGCGGTTCCTCTCCGTGGGCTACGTGGACGAT
CAGCAGTTCGTGCGCTTCGACAGCGACAGCGCGAGTCAGAGTGAGGAGCCGCGGGCGCCG

TGGATAGAGAAGGTGCAGGACGTGGACCCGGGATACTGGGAGCGGAACACACAGATCAGT
AAGGAGAACGCACAGAGTTCCCGAGTGAGCCTGCAGAACCTGCGC
>SahaI*31
GCCGTGTCCCGGCCCGGGCTCGGGGAGCCGCGGTTCCTCTCCGTGGGCTACGTGGACGAT
CAGCAGTTCGTGCGCTTCGACAGCGACAGCGCGAGTCAGAGTGAGGAGCCGCGGGCGCCG
TGGATGGAGAAGGTGCAGGACGTGGACCCGGGATACTGGGAGCGGAACACGCAGATCAGT
AAGGAGAACGCACAGAATTCCCGAGTGAGCCTGCAGAACCTGCGC
>SahaI*33
GCCGTGTCCCGGCCCGGGCTCGGGGAGCCGCGGTTCCTCTCCGTGGGCTACGTGGACGAT
CAGCAGTTCGTGCGCTTCGACAGCGACAGCGCGAGTCAGAGTGAGGAGCCGCGGGCGCCG
TGGATGGAGAAGGTGAAGGACGTGGACCCGGGATACTGGGAGCAGCAGACACAGATCATT
AAGGAGACTGCACAGATTTACCGAGTGGGCCTGCAGACCCTGCGC
>SahaI*34
GCCGTGTCCCGGCCCGGGCTCGGGGAGCCGCGGTTCCTCTCCGTGGGCTACGTGGACGAT
CAGCAGTTCGTGCGCTTCGACAGCGACAGCGCGAGTCAGAGTGAGGAGCCGCGGGCGCCG
TGGATGGAGAAGGTGAAGGACGTGGACCCGGGATACTGGGAGCAGCAGACACAGATCATT
AAGGAGACTGCACAGATTTCCCGAGTGGACCTGCAGACCCTGCGC
>SahaI*36
GCCGTGTCCCGGCCCGGGCTCGGGGAGCCGCGGTTCCTCTCCGTGGGCTACGTGGACGAT
CAGCAGTTCGTGCGCTTCGACAGCGACAGCGCGAGTCAGAGTGAGGAGCCGCGGGCGCCG
TGGATGGAGAAGGTGAAGGACGTGGACCCGGGATACTGGGAGCAGGAGACACAGATCAGT
AAGGAGACTGCACAGATTTACCGAGTGGGCCTGCAGACCCTGCGC
>SahaI*37
GCCGTGTCCCGGCCCGGGCTCGGGGAGCCGCGGTTCCTCGCCGTGGGCTACGTGGACGAT
CAGCAGTTCGTGCGCTTCGACAGCGACAGCGCGAGTCAGAGTGAGGAGCCGCGGGCGCCG
TGGATGGAGAAGGTGAAGGACGTGGACCCGGGATACTGGGAGCAGGAGACACAGATCAGT
AAGGAGAACGCACAGATTTACCGAGTGGGCCTGCAGACCCTGCGC
>SahaI*38
GCCGTGTCCCGGCCCGGGCTCGGGGAGCCGCGGTTCCTCGCCGTGGGCTACGTGGACGAT
CAGCAGTTCGTGCGCTTCGACAGCGACAGCGCGAGTCAGAGTGAGGAGCCGCGGGCGCCG
TGGATGGAGAAGGTGCAGGACGTGGACCCGGGATACTGGGAGCAGGAGACACAGATCATT
AAGGAGACTGCACAGATTTCCCGAGTGGACCTGCAGACCCTGCGC
>SahaI*39
ACCGTGTCCCGGCCCGGACTCGGGGAGCCGCGATTCTTCTCCGTGGGCTACGTGGACGAT
CAGCAGTTCGTGGGCTTCGACAGCGACAGTGCGAGTCAGAGGGTGGAGCCGCGGGCACCA
TGGATAGAGAAGATGGAGAATGTGGACCGGGACTACTGGGAGCGGAACACGCAGAACAGT
AAGAGGAACGCACAAATTTCCCGAGAGGACCTGCAGACCCTACAC
>SahaI*40
ACCGTGTCCCGGCCCGGACTCGGGGAGCCGCGATTCTTCTCCGTGGGCTACGTGGACGAT
CAGCAGTTCGTGGGCTTCGACAGCGACAGTGCGAGTCAGAGGGTGGAGCCGCGGGCACCA
TGGATAGAGAAGATGGAGAATGTGGACCGGGACTACTGGGAGCGGAACACGCAGAACAGT
AAGAGGAATGCACAAACTTCCCGAGAGGACCTGCAGACCCTACAC
>SahaI*41
ACCGTGTCCCGGCCCGGGCTCGGGGAGCCGCGATTCTTCTCCGTGGGCTACGTGGACGAT
CAGCAGTTCGTGGGCTTCGACAGCGACAGTGCGAGTCAGAGGGTGGAGCCGCGGGCACCA
TGGATAGAGAAGATGGAGAATGTGGACCGGGACTACTGGGAGCGGAACACGCAGAACAGT
AAGAGGAATGCACAAATTTCCCGAGAGGACCTGCAGACCCTACAC
>SahaI*42
ACCGTGTCCCGGCCCGGGCTCGGGGAGCCGCGGTTCTTCTCCGTGGGCTACGTGGACGAT
CAGCAGTTCGTGGGCTTCGACAGCGACAGTGCGAGTCAGAGGGTGGAGCCGCGGGCACCA
TGGATAGAGAAGATGGAGAATGTGGACCGGGACTACTGGGAGCGGAACACGCAGAACAGT
AAGAGGAATGCACAAATTTCCCGAGAGGACCTGCAGACCCTACAC
>SahaI*43
ACCGTGTCCCGGCCCGGACTCGGGGAGCCGCGATTCTTCTCCGTGGGCCACGTGGACGAT
CAGCAGTTCGTGGGCTTCGACAGCGACAGTGCGAGTCAGAGGGTGGAGCCGCGGGCACCA
TGGATAGAGAAGATGGAGAATGTGGACCGGGACTACTGGGAGCGGAACACGCAGAACAGT
AAGGGGAATGCACAAATTTCCCGAGAGGACCTGCAGACCCTACAC
>SahaI*44

GCCGTGTCCCGGCCCGGGCTCGGGGAGCCGCGGTTCCTCGCCGTGGGCTACGTGGACGAT
CAGCAGTTCGTGCGCTTCGACAGCGACAGCGCGAGTCAGAGGCAGGAGCCGCGGGCGCCG
TGGATGGAGAAGGTGAAGGACGTGGACCCGGGATACTGGGAGCAGCAGACACAGATCAGT
AAGGAGAACGCACAGATTTACCGAGTGGGCCTGCAGACCCTGCGC
>SahaI*45
GCCGTGTCCCGGCCCGGGCTCGGGGAGCCGCGGTTCCTCTCCGTGGGCTACGTGGACGAT
CAGCAGTTCGTGCGCTTCGACAGCGACAGCGCGAGTCAGAGTGAGGAGCCGCGGGCGCCG
TGGATGGAGAAGGTGCAGGACGTGGACCCGGGATACTGGGAGCAGGAGACACAGATCATT
AAGGAGAACGCACAGAGTTCCCGAGTGGACCTGCAGACCCTGCGC
>SahaI*47
GCCGTGTCCCGGCCCGGGCTCGGGGAGCCGCGGTTCCTCGCCGTGGGCTACGTGGACGAT
CAGCAGTTCGTACGCTTCGACAGCGACAGCGCGAGTCAGAGTGAGGAGCCGCGGGCGCCG
TGGATGGAGAAGGTGCAGGACGTGGACCCGGGATACTGGGAGCAGCAGACACAGAACAGT
AAGGGGAATGCACAGATTTACCGAGTGGGCCTGCAGACCCTGCGC
>SahaI*48
GCCGTGTCCCGGCCCGGGCTCGGGGAGCCGCGGTTCCTCGCCGTGGGCTACGTGGACGAT
CAGCAGTTCGTGCGCTTCGACAGCGACAGCGCGAGTCAGAGTGAGGAGCCGCGGGCGCCG
TGGATGGAGAAGGTGCAGGACGTGGACCCGGGATACTGGGAGCAGGAGACACAGATCATT
AAGGAGAACGCACAGAGTTCCCGAGTGAGCCTGCAGAACCTGCGC
>SahaI*49
GCCGTGTCCCGGCCCGGGCTCGGGGAGCCGCGGTTCCTCGCCGTGGGCTACGTGGACGAT
CAGCAGTTCGTGCGCTTCGACAGCGACAGCGCGAGTCAGAGTGAGGAGCCGCGGGCGCCG
TGGATGGAGAAGGTGAAGGACGTGGACCCGGGATACTGGGAGCAGCAGACACAGATCAGT
AAGGAGAACGCACAGATTTACCGAGTGGGCCTGCAGACCCTGCGC
>SahaI*50
GCCGTGTCCCGGCCCGGGCTCGGGGAGCCGCGGTTCCTCTCCGTGGGCTACGTGGACGAT
CAGCAGTTCGTGCGCTTCGACAGCGACAGCGCGAGTCAGAGTGAGGAGCCGCGGGCGCCG
TGGATGGAGAAGGTGAAGGACGTGGACCCGGGATACTGGGAGCAGGAGACACAGATCATT
AAGGAGAACGCACAGAGTTCCCGAGTGGACCTGCAGACCCTGCGC
>SahaI*51
GCCGTGTCCCGGCCCGGGCTCGGGGAGCCGCGGTTCCTCGCCGTGGGCTACGTGGACGAT
CAGCAGTTCGTGCGCTTCGACAGCGACAGCGCGAGTCGGAGTGAGGAGCCGCGGGCGCCG
TGGATGGAGAAGGTGCAGGACGTGGACCCGGGATACTGGGAGCAGGAGACACAGATCATT
AAGGAGAACGCACAGAGTTCCCGAGTGGACCTGCAGACCCTGCGC
>SahaI*52
GCCGTGTCCCGGCCCGGGCTCGGGGAGCCGCGGTTCCTCTCCGTGGGCTACGTGGACGAT
CAGCAGTTCGTGCGCTTCGACAGCGACAGCGCGAGTCAGAGTGAGGAGCCGCGGGCGCCG
TGGATGGAGAAGGTGAAGGACGTGGACCCGGGATACTGGGAGCAGGAGACACAGATCGTT
AAGGAGACTGCACAGATTTCCCGAGTGGACCTGCAGACCCTGCGC
>SahaI*53
GCCGTGTCCCGGCCCGGGCTCGGGGAGCCGCGGTTCCTCTCCGTGGGCTACGTGGACGAT
CAGCAGTTCGTGCGCTTCGACAGCGACAGCGCGAGTCAGAGTGAGGAGCCGCGGGCGCCG
TGGATGGAGAAGGTGAAGGACGTGGACCCGGGATACTGGGAGCGGAACACACAGATCAGT
AAGGAGAACGCACAGAGTTCCCGAGTGAGCCTGCAGACCCTGCGC
>SahaI*54
GCCGTGTCCCGGCCCGGGCTCGGGGAGCCGCGGTTCCTCGCCGTGGGCTACGTGGACGAT
CAGCAGTTCGTGCGCTTCGACAGCGACAGCGCGAGTCAGAGTGAGGAGCCGCGGGCGCCG
TGGATGGAGAAGGTGAAGGACGTGGACCCGGGATACTGGGAGCGGAACACACAGATCAGT
AAGGAGAACGCACAGAGTTCCCGAGTGAGCCTGCAGACCCTGCGC
>SahaI*55
ACCGTGTCCCGGCCCGGACTCGGGGAGCCGCGATTCTTCTCCGTGGGCTACGTGGACGAT
CAGCAGTTCGTGGGCTTCGACAGCGACAGTGCGAGTCAGAGGGTGGGGCCGCGGGCACCA
TGGATAGAGAAGATGGAGAATGTGGACCGGGACTACTGGGAGCGGAACACGCAGAACAGT
AAGAGGAATGCACAAATTTCCCGAGAGGACCTGCAGACCCTACAC
>SahaI*56
GCCGTGTCCCGGCCCGGGCTCGGGGAGCCGCGGTTCCTCTCCGTGGGCTACGTGGACGAT
CAGCAGTTCGTGCGCTTCGACAGCGACAGCGCGAGTCAGAGTGAGGAGCCGCGGGCGCCG
TGGATGGCGAAGGTGCAGGACGTGGACCCGGGATACTGGGAGCGGAACACACAGATCAGT

AAGGAGAACGCACAGAGTTCCCGAGTGAGCCTGCAGAACCTGCGC
>SahaI*57
GCCGTGTCCCGGCCCGGGCTCGGGGAGCCGCGGTTCCTCGCCGTGGGCTACGTGGACGAT
CAGCAGTTCGTGCGCTTCGACAGCGACAGCGCGAGTCAGAGTGAGGAGCCGCGGGCGCCG
TGGATGGAGAAGGTGCAGGACGTGGACCCGGGATACTGGGAGCGGAACACACAGATCAGT
AAGGAGAACGCACAGAGTTCCCGAGTGGACCTGCAGACCCTGCGC
>SahaI*58
GCCGTGTCCCGGCCCGGGCTCGGGGAGCCGCGGTTCCTCGCCGTGGGCTACGTGGACGAT
CAGCAGTTCGTGCGCTTCGACAGCGACAGCGCGAGTCAGAGTGAGGAGCCGCGGGCGCCG
TGGATGGAGAAGGTGCAGGACGTGGACCCGGAATACTGGGAGCGGAACACACAGATCAGT
AAGGAGAACGCACAGAGTTCCCGAGTGGACCTGCAGACCCTGCGC
>SahaI*59
GCCGTGTCCCGGCCCGGGCTCGGGGAGCCGCGGTTCCTCTCCGTGGGCTACGTGGACGAT
CAGCAGTTCGTGCGCTTCGACAGCGACAGCGCGAGTCAGAGTGAGGAGCCGCGGGCGCCG
TGGATGGAGAAGATGAAGGACGTGGACCCGGGATACTGGGAGCAGGAGACACAGATCAGT
AAGGAGAATGCACAGATTTCCCGAGTGGACCTGCAGACCCTGCGC
>SahaI*60
ACCGTGTCCCGGCCCGGACTCAGGGAGCCGCGATTCTTCTCCGTGGGCTACGTGGACGAT
CAGCAGTTCGTGGGCTTCGACAGCGACAGTGCGAGTCAGAGGGTGGAGCCGCGGGCACCA
TGGATAGAGAAGATGGAGAATGTGGACCGGGACTACTGGGAGCGGAACACGCAGAACAGT
AAGAGGAATGCACAAATTTCCCGAGAGGACCTGCAGACCCTACAC
>SahaI*61
GCCGTGTCCCGACCCGGGCTCGGGGAGCCGCGGTTCCTCTCCGTGGGCTACGTGGACGAT
CAGCAGTTCGTGCGCTTCGACAGCGACAGCGCGAGTCAGAGTGAGGAGCCGCGGGCGCCG
TGGATGGAGAAGGTGCAGGACGTGGACCCGGGATACTGGGAGCGGAACACACAGATCAGT
AAGGAGAACGCACAGAGTTCCCGAGTGAGCCTGCAGAACCTGCGC
>SahaI*62
GCCGTATCCCGGCCCGGGCTCGGGGAGCCGCGGTTCCTCTCCGTGGGCTACGTGGACGAT
CAGCAGTTCGTGCGCTTCGACAGCGACAGCGCGAGTCAGAGTGAGGAGCCGCGGGCGCCG
TGGATGGAGAAGGTGCAGGACGTGGACCCGGGATACTGGGAGCGGAACACACAGATCAGT
AAGGAGAACGCACAGAGTTCCCGAGTGAGCCTGCAGAACCTGCGC
>SahaI*63
GCCGTGTCCCGGCCCGGGCTCGGGGAGCCGCGGTTCCTCGCCGTGGGCTACGTGGACGAT
CAGCAGTTCGTGCGCTTCGACAGCGACAGCGCGAGTCAGAGTGAGGAGCCGCGGGCGCCG
TGGATGGAGAAGGTGCAGGACGTGGACCCGGGATACTGGGAGCAGGAGACACAGATCATT
AAGGAGAACGCACAGAGTTCTCGAGTGGACCTGCAGACCCTGCGC
>SahaI*65
GCCGTGTCCCGGCCCGGGCTCGAGGAGCCGCGGTTCCTCTCCGTGGGCTACGTGGACGAT
CAGCAGTTCGTGCGCTTCGACAGCGACAGCGCGAGTCAGAGTGAGGAGCCGCGGGCGCCG
TGGATGGAGAAGGTGCAGGACGTGGACCCGGGATACTGGGAGCGGAACACACAGATCAGT
AAGGAGAACGCACAGAGTTCCCGAGTGAGCCTGCAGAACCTGCGC
>SahaI*66
GCCGTGTCCCGGCCCGGGCTCGGGGAGCCGCGATTCCTCTCCGTGGGCTACGTGGACGAT
CAGCAGTTCGTGCGCTTCGACAGCGACAGCGCGAGTCAGAGTGAGGAGCCGCGGGCGCCG
TGGATGGAGAAGGTGCAGGACGTGGACCCGGGATACTGGGAGCAGGAGACACAGATCATT
AAGGAGAACGCACAGAGTTCCCGAGTGGACCTGCAGACCCTGCGC
>SahaI*67
GCCGTGTCCCGGCCCGGGCTCGGGGAGCCGCGGTTCCTCTCCGTGGGCTACGTGGACGAT
CAGCAGTTCGTGGGCTTCGACAGCGACAGTGCGAGTCAGAGGGTGGAGCCGCGGGCACCA
TGGATAGAGAAGATGGAGAATGTGGACCGGGACTACTGGGAGCGGAACACGCAGAACAGT
AAGAGGAATGCACAAATTTCCCGAGAGGACCTGCAGACCCTACAC
>SahaI*68
GCCGTGTCCCGGCCCGGGCTCGGGGAGCCGCGGTTCCTCGCCGTGGGCTACGTGGACGAT
CAGCAGTTCGTGCGCTTCGACAGCGACAGCGCGAGTCAGAGTGAGGAGCCGCGGGTGTCG
TGGATGGAGAAGGTGCAGGACGTGGACCCGGGATACTGGGAGCGGAACACACAGATCAGT
AAGGAGAACGCACAGAGTTCCCGAGTGAGCCTGCAGAACCTGCGC
>SahaI*69
GCCGTGTCCCGGCCCGGGCTCGGGGAGCCGCGATTCCTCTCCGTGGGCTACGTGGACGAT

CAGCAGTTCGTGCGCTTCGACAGCGACAGCGCGAGTCAGAGTGAGGAGCCGCGGGCGCCG
TGGATGGAGAAGGTGCAGGACGTGGACCCGGGATACTGGGAGCAGGAGACACAGATCATT
AAGGAGACTGCACAGATTTCCCGAGTGGACCTGCAGACCCTGCGC
>SahaI*70
GCCGTGTCCCGGCCTGGGCTCGGGGAGCCGCGGTTCCTCTCCGTGGGCTACGTGGACGAT
CAGCAGTTCGTGCGCTTCGACAGCGACAGCGCGAGTCAGAGTGAGGAGCCGCGGGCGCCG
TGGATGGAGAAGGTGCAGGACGTGGACCCGGGATACTGGGAGCAGGAGACACAGATCATT
AAGGAGACTGCACAGATTTCCCGAGTGGACCTGCAGACCCTGCGC
>SahaI*71
ACCGTGTCCCGGCCCGGACTCGGGGAGCCGCGATTCTTCTCCGTGGGCTACGTGGACGAT
CAGCAGTTCGTGGGCTTCGACAGCGACAGTGCGAGTCAGAGGGTGGAGCCGCGGGCACCA
TGGATAGAGAAGATGGAGAATATGGACCGGGACTACTGGGAGCGGAACACGCAGAACAGT
AAGAGGAATGCACAAATTTCCCGAGAGGACCTGCAGACCCTACAC
>SahaI*72
GCCGTGTCCCGGCCCGGACTCGGGGAGCCGCGGTTCCTCGCCGTGGGCTACGTGGACGAT
CAGCAGTTCGTGCGCTTCGACAGCGACAGCGCGAGTCAGAGTGAGGAGCCGCGGGCGCCG
TGGATGGAGAAGGTGAAGGACGTGGACCCGGGATACTGGGAGCAGGAGACACAGATCAGT
AAGGAGAACGCACAGATTTACCGAGTGGGCCTGCAGACCCTGCGC
>SahaI*73
ACCGTGTCCCGGCCCGGACTCGGGGAGCCGCGATTCTTCTCCGTGGGCTACGTGGACGAT
CAGCAGTTCGTGGGCTTCGACAGCGACAGTGCGAGTCAGAGGGTGGAGCCGCGGGCACCA
TGGATAGAGAAGATGGAGAATGTGGACCGGGACTACTGGGAGCGGAACACGCAGAACAGT
AAGAGGAATGCACAAATTTCCCGAGGGGACCTGCAGACCCTACAC
>SahaI*74
GCCGTGTCCCGGCCCGGGCTCGGGGAGCCGCGGTTCCTCGCCGTGGGCTACGTGGACGAT
CAGCAGTTCGTGCGCTTCGACAGCGACAGCGCGAGTCAGAGTGAGGAGCCGCGGGCGCCG
TGGATGGAGAAGGTGCAGGACGTGGACCCGGGATACTGGGAGCGGAACACACAGATCAGT
AAGGAGAACGCACAGAGTTCCCGAGTGAGCCTGCAGACCCTGCGC
>SahaI*75
ACCGTGTCCCGGCCCGGACTCGGGGAGCCGCGATTCTTCTCCGTGGGCTACGTGGACGAT
CAGCAGTTCGTGGGCTTCAACAGCGACAGTGCGAGTCAGAGGGTGGAGCCGCGGGCACCA
TGGATAGAGAAGATGGAGAATGTGGACCGGGACTACTGGGAGCGGAACACGCAGAACAGT
AAGAGGAATGCACAAATTTCCCGAGAGGACCTGCAGACCCTACAC
>SahaI*76
ACCGTGTCCCGGCCCGGACTCGGGGAGCCGCGATTCTTCTCCGTGGGCTACGTGGACGAT
CAGCAGTTCGTGGGCTTCGACAGCGACAGTGCGAGTCAGAGGGTGGAGCCGCGGGCACCA
TGGATAGAGAAGATGGAGAATGTGGACCGGGACTACTGGGAGCGGAACACGCAGAACAGT
AAGAGGAATGCACGAATTTCCCGAGAGGACCTGCAGACCCTACAC
>SahaI*77
ACCGTGTCCCGGCCCGGACTCGGGGAGCCGCGATTCTTCTCCGTGGGCTACGTGGACGAT
CAGCAGTTCGTGGGCTTCGACAGCGACAATGCGAGTCAGAGGGTGGAGCCGCGGGCACCA
TGGATAGAGAAGATGGAGAATGTGGACCGGGACTACTGGGAGCGGAACACGCAGAACAGT
AAGAGGAATGCACAAATTTCCCGAGAGGACCTGCAGACCCTACAC
>SahaI*78
GCCGTGTCCCGGCCCGGGCTCGGGGAGCCGCGGTTCTTCGCCGTGGGCTACGTGGACGAT
CAGCAGTTCGTGCGCTTCGACAGCGACAGCGCGAGTCAGAGTGAGGAGCCGCGGGCGCCG
TGGATGGAGAAGGTGCAGGACGTGGACCCGGGATACTGGGAGCGGAACACACAGATCAGT
AAGGAGAACGCACAGAGTTCCCGAGTGAGCCTGCAGAACCTGCGC
>SahaI*79
GCCGTGTCCCGGCCCGGGCTCGGGGAGCCGCGGTTCCTCTCCGTGGGCTACGTGGACGAT
CAGCAGTTCGTGCGCTTCGACAGCGACAGCGCGAGTCAGAGTGAGGAGCCGCGGGCGCCG
TGGATGGAGAAGGTGAAGGACGTGGACCCGGGATACTGGGAGCAGGAGACACAGATCAGT
AAGGAGAACGCACAGATTTACCGAGTGGGCCTGCAGACCCTGCGC
>SahaI*80
GCCGTGTCCCGGCCCGGGCTCGGGGAGCCGCGGTTCCTCTCCGTGGGCTACGTGGACGAT
CAGCAGTTCGTGCGCTTCGACAGCGACAGCGCGAGTCAGAGTGAGGAGCCGCAGGCGCCG
TGGATGGAGAAGGTGAAGGACGTGGACCCGGGATACTGGGAGCAGGAGACACAGATCAGT
AAGGAGAACGCACAGAGTTCCCGAGTGAGCCTGCAGAACCTGCGC

```
>SahaI*82
GCCGTGTCCCGGCCCGGGCTCGGGGAGCCGCAGTTCCTCGCCGTGGGCTACGTGGACGAT
CAGCAGTTCGTGCGCTTCGACAGCGACAGCGCGAGTCAGAGTGAGGAGCCGCGGGCGCCG
TGGATGGAGAAGGTGAAGGACGTGGACCCGGGATACTGGGAGCAGCAGACACAGATCAGT
AAGGAGAACGCACAGATTTACCGAGTGGGCCTGCAGACCCTGCGC
>SahaI*83
ACCGTGTCCCGGCCCGGACTCGGGGAGCCGCGATTCTTCTCCGTGGGCTACGTGGACGAT
CAGCAGTTCGTGGGCTTCGACAGCGATAGTGCGAGTCAGAGGGTGGAGCCGCGGGCACCA
TGGATAGAGAAGATGGAGAATGTGGACCGGGACTACTGGGAGCGGAACACGCAGAACAGT
AAGAGGAATGCACAAATTTCCCGAGAGGACCTGCAGACCCTACAC
>SahaI*84
ACCGTGTCCCGGCCCGGACTCGGGGAGCCGCGATTCTTCTCCGTGGGCTACGTGGACGAT
CAGCAGTTCGTGGGCTTCGACGGCGACAGTGCGAGTCAGAGGGTGGAGCCGCGGGCACCA
TGGATAGAGAAGATGGAGAATGTGGACCGGGACTACTGGGAGCGGAACACGCAGAACAGT
AAGAGGAATGCACAAATTTCCCGAGAGGACCTGCAGACCCTACAC
>SahaI*85
ACCGTGTCCCGGCCCGGACTCGGGGAGCCGCGATTCTTCTCCGTGGGCTACGTGGACGAT
CAGCAGTTCGTGGGCTTCGACAGCGACAGTGCAAGTCAGAGGGTGGAGCTGCGGGCACCA
TGGATAGAGAAGATGGAGAATGTGGACCGGGACTACTGGGAGCGGAACACGCAGAACAGT
AAGAGGAATGCACAAATTTCCCGAGAGGACCTGCAGACCCTACAC
>SahaUK
TCCATGTCTCAGCCCGGGCTCACAAAGCCGAGGTTCATTTCGGTGGGCTATGTGGACGAT
CAGGCGTTCGTTCGCTTCGACAGCGACAGCGCAGGTCTGAGGGAGGAGCCCATGGTGATG
TGGTTGGACAAGAGTAACCAGAATTACTGGGAGAGGAACTCGCGTGTCATCTGGGAGACT
GCACGCACTTTCCAAGTGGGCCTGCAGAATCTGCAGTTCTATTAC
>ModoUK
TCCATGTCTCTGCCTGGG---AGAAAGCCGCAGTTCATCTCTGTGGGCTACGTGGACGAT
CTGCAGTTCATGCTCTTTGACAGGAACAGCCCGAATCAGAGGGAGGAGCCCAGAGCACCG
TGGTTGGACCAGATGGACCAGGATTACTGGGAGAAGAACTCAAGAATTTCCAGGGAGACC
GCTCAGACTTTCGAAGTGGGCCTGCAGAATCTGCTGGTCTATTAC
>MaeuUK
TCCATGTCTCTCTCCGGGCTTGCAAAGCCGAGGTTCATTTCTGTGGGTTACGTGGACGAT
CAGCAGTTTGTACGCTTTGACAGCGACAACTGGAGTCAGAGGGAAGAGCCCCAGGCACCT
TGGGTGGACAAGATCAGTCAGGAGGACTGGGCAAGGAACTCACGAGTAGTTAGAGAGACG
GAGCACACTTTCCAAGTGGGCCTGCAGAATCTGCAAGTCTATTAC
>ModoUB
GCCATGTCCCGGCCTGAGCTCGGGGACTCCCAGTTCATCACGGTGGGCTACGTGGACGAT
CAGCAGTTCGTGCGCTTCGACAGCAGCAGCGAGAGTCAGAGGATGGAGCCTCGGGCGGCC
TGGATGGACAAGATGGAGGAGGAGGAACCGAACTACTGGGAGGAGCAGACGCAGATCTAC
AGGAAGAACGCGCAGATTTACCAAAGGAACCTGGAGACCCTGCGC
>ModoUC
GCCATGTCCCGGCCTGAGCTCGGGGACTCCCAGTTCATCACGGTGGGCTACGTGGACGAT
CAGCAGTTCGTGCGCTTCGACAGCAGCAGCGAGAGTCAGAGGATGGAGCCGCGGGCGGCC
TGGATGGACAAGATGGAGGAGGAGGAACCGAACTACTGGGAGGAGCAGACGCAGATCTAC
AGGAAGAACGCGCAGATTTACCGAGGGAACCTGGAGACCCTGCGC
>ModoUA
GCCATGTCCCGGCCTGAGCTCGGGGACTCGCAGTTCATCTCGGTGGGCTACGTGGACGAT
CAGCAGTTTGTGCGCTTCGACAGCAGCAGCGAGAGTCCGAGGATGGAGCCCCGGGCGGCC
TGGATGGACAAGGTGGACCAGGAGGACCCGAACTACTGGGAGGGGCAGACGCAGATCTCC
AGGAGCAATGCACAGATTACCAGAGTGGGCCTGGAGACCATTCGA
```
